# Association Between Systolic Blood Pressure at Emergency Department Arrival and Time to Bleeding Cessation in Adult Non-Traumatic Epistaxis: A Retrospective Time-to-Event Analysis

**DOI:** 10.3390/jcm15145535

**Published:** 2026-07-15

**Authors:** Phawinee Phiromkanchanasak, Suwijahk Chaiphrom, Bhumsapanan Thiamkaew, Wannagon Thanadolyothin, Wachira Wongtanasarasin

**Affiliations:** 1 Department of Emergency Medicine, Faculty of Medicine, Chiang Mai University, Chiang Mai 50200, Thailand; phawinee_phiromkan@cmu.ac.th (P.P.);; 2 Acute Care and Emergency Medicine (ACE) Research Cluster, Faculty of Medicine, Chiang Mai University, Chiang Mai 50200, Thailand

**Keywords:** epistaxis, blood pressure, hypertension, emergency department, hemostasis, time-to-event analysis

## Abstract

**Objectives:** Epistaxis is a common emergency department (ED) presentation, but the prognostic significance of triage blood pressure (BP) remains unclear. We evaluated whether elevated systolic BP (SBP) at ED arrival was associated with delayed bleeding cessation in adults with non-traumatic epistaxis. **Methods:** We conducted a retrospective cohort study of adults (≥18 years) presenting with non-traumatic epistaxis to a tertiary ED in Northern Thailand between 2015 and 2025. Patients with documented triage SBP were included (*n* = 482). The exposure was SBP at ED arrival, categorized as ≥140 versus <140 mmHg. The primary outcome was time from ED arrival to first documented bleeding cessation. Adjusted hazard ratios (aHRs) were estimated using Cox proportional hazards regression, controlling for demographics, comorbidities, medication use, and ED interventions. **Results:** Among 482 patients, 310 (64.3%) had SBP ≥ 140 mmHg. Median time to documented bleeding cessation was longer in patients with elevated SBP than in those with SBP < 140 mmHg (39 vs. 23 min). In adjusted analyses, SBP ≥ 140 mmHg was associated with delayed documented bleeding cessation (aHR, 0.71; 95% CI, 0.52–0.98). Mechanical nasal packing was also associated with slower bleeding control (aHR, 0.72; 95% CI, 0.54–0.95), whereas electrical cauterization was associated with faster cessation (aHR, 2.17; 95% CI, 1.19–3.96). **Conclusions:** Elevated SBP at ED arrival was associated with prolonged time to documented bleeding cessation in non-traumatic epistaxis. Triage SBP may serve as a pragmatic early prognostic marker of prolonged ED management. However, important bleeding severity characteristics, including bleeding location and estimated blood loss, were unavailable, and residual confounding cannot be excluded. These findings should not be interpreted as evidence supporting acute antihypertensive therapy to accelerate hemostasis. Prospective studies are needed to validate these findings.

## 1. Introduction

Epistaxis is among the most common otolaryngologic emergencies, with an estimated lifetime prevalence of up to 60% and accounting for approximately 1 in 200 emergency department (ED) visits [1]. Although most cases resolve spontaneously, a clinically significant subset requires active intervention such as packing, cautery, or topical hemostatic agents [2,3]. Prolonged or recurrent bleeding can increase ED length of stay and resource utilization [4,5].

Whether blood pressure (BP) contributes to the severity or duration of epistaxis is debated [6]. Prior studies report conflicting associations between elevated BP, hypertension history, and severity or persistence of bleeding [3,7,8]. Some investigations suggest that higher systolic or diastolic BP is linked to more difficult-to-control epistaxis and increased need for intervention. In contrast, others report no independent association [3,9,10,11,12,13]. Existing analyses typically evaluate binary outcomes rather than characterize the time course of bleeding control [7,14].

Time-to-event approaches better characterize bleeding cessation over time than binary outcome measures [15,16]. Despite this advantage, the prognostic value of triage BP—an immediately available physiologic marker—has not been well evaluated using time-to-event methods. Understanding whether elevated systolic BP at presentation is associated with delayed bleeding cessation may help clinicians anticipate prolonged courses and tailor early management. This study examined the association between systolic BP at ED arrival and time to documented bleeding cessation among adults with non-traumatic epistaxis.

## 2. Materials and Methods

### 2.1. Study Design and Setting

We conducted a retrospective observational cohort study at the Emergency Department of Maharaj Nakorn Chiang Mai Hospital, a large tertiary referral center in Northern Thailand. The study included all adult presentations for epistaxis between January 2015 and March 2025. The protocol was approved by the Institutional Review Board of the Faculty of Medicine, Chiang Mai University, which waived the requirement for informed consent due to the minimal risk associated with the retrospective design. Patient data were anonymized and handled in compliance with the Declaration of Helsinki and local data protection regulations. During the preparation of this work, the authors used ChatGPT 5.1 and Grammarly (https://www.grammarly.com/) in order to improve language clarity. After using this tool/service, the authors reviewed and edited the content as needed and take full responsibility for the published article.

### 2.2. Case Identification and Eligibility

Patients with ICD-10 code R04.0 (epistaxis) were identified from electronic health records (EHR). Trauma-related epistaxis and life-threatening presentations requiring immediate cardiopulmonary intervention were excluded. Systolic BP at ED arrival was required for inclusion. Trauma exclusions were verified via accompanying trauma-specific ICD codes and clinician notes.

### 2.3. Exposure, Outcome, and Data Extraction

Demographics, comorbidities, outpatient medications, ED vital signs, and management interventions were extracted from structured fields in the electronic health record. Comorbidities were defined using ICD-10 codes and, when applicable, confirmed by medication lists. Anticoagulant and antiplatelet use reflected pre-arrival outpatient regimens recorded during triage.

Bleeding cessation was documented using structured clinician-defined fields (“bleeding stopped,” “no further bleeding,” or equivalent predefined entries). When multiple assessments were present, the earliest documented cessation time was used. No natural language processing was used.

The primary exposure was systolic blood pressure at ED arrival. Triage SBP was measured by automated oscillometric devices operated by trained triage nurses in a seated position. The initial recorded value was used. Systolic BP was categorized as <140 vs. ≥140 mmHg based on widely used thresholds in prior epistaxis literature and hypertension guidelines [7].

The primary outcome was time from ED arrival to the first documented assessment indicating cessation of bleeding. Bleeding cessation was defined according to structured clinician documentation fields (e.g., “bleeding stopped,” “no further bleeding,” or equivalent predefined entries). Because continuous monitoring of bleeding status was unavailable in this retrospective dataset, the recorded time reflects the first documented confirmation of hemostasis rather than the exact physiological moment when bleeding ceased.

Patients without documented bleeding cessation before ED discharge were treated as right-censored observations at the time of their last documented clinical assessment in the emergency department. These patients contributed follow-up time until censoring but were not assumed to have achieved hemostasis. The number of censored observations is reported in Section 3.

### 2.4. Interventions

Management strategies within the first hour of ED care were categorized into composite variables: (1) local hemostatic agents include oxymetazoline spray, oxidized cellulose (SURGICEL^®^, Ethicon Inc., Somerville, NJ, USA), gelatin foam (Gelfoam^®^, Pfizer Inc., Kalamazoo, MI, USA), calcium–sodium alginate (Kaltostat^®^, ConvaTec Ltd., Deeside, UK), and tranexamic acid; (2) mechanical packing includes rolled gauze, nasal tampons, and a nasal balloon catheter; and (3) electrical cauterization. Interventions were identified from structured order sets and procedure fields.

### 2.5. Sample Size Estimation and Statistical Analysis

The required sample size was estimated using the Schoenfeld method for Cox proportional hazards models, which is based on the number of outcome events rather than the total number of participants [17]. Previous studies have suggested that elevated BP is associated with a more severe clinical course or prolonged bleeding in patients with epistaxis [3,10,11]. Reported effect sizes range from modest to moderate, with odds ratios or hazard ratios of 1.33 for persistent bleeding or severe presentations among patients with higher blood pressure [11] along with a two-sided α of 0.05, and 80% power, with an exposure prevalence of 50% (proportion of patients arriving with elevated blood pressure). This yielded a requirement of approximately 386 events. Because bleeding cessation occurred in most patients, we assumed an event proportion of 0.85 based on prior ED cohorts [10,11]. Thus, the minimum required total sample size was 455 patients.

Continuous variables were summarized as means ± standard deviations or medians with interquartile ranges, depending on distribution. Categorical variables were presented as counts and percentages. Baseline differences were compared using Student’s *t*-test or Mann–Whitney *U* test for continuous variables and chi-square or Fisher’s exact test for categorical variables. Standardized differences (STDs) were additionally calculated for baseline variables to quantify the magnitude of between-group differences independent of sample size.

For time-to-event analysis, survival data were declared using time to documented bleeding cessation as the time variable and hemostasis as the event indicator. Kaplan–Meier curves were generated to estimate time to hemostasis, stratified by blood pressure categories (SBP at ED arrival <140 mmHg vs. ≥140 mmHg), and compared using the log-rank test.

Cox proportional hazards regression was then used to evaluate the association between SBP category and time to documented bleeding cessation. Results were expressed as hazard ratios (HRs) with 95% confidence intervals (CIs), where an HR > 1 indicates a higher hazard of documented bleeding cessation (i.e., a shorter time to documented bleeding cessation), whereas an HR < 1 indicates a lower hazard of documented bleeding cessation (i.e., a longer time to documented bleeding cessation). Multivariable models adjusted for age, sex, hypertension history, anticoagulant/antiplatelet use, bleeding and vascular disorders, history of recent epistaxis within 72 h prior to arrival, inhaled corticosteroid use, and interventions given to the patients. A total of 482 patients were included in the Cox regression analyses, of whom 447 achieved documented cessation of bleeding during follow-up. The remaining patients were censored at the time of ED disposition or the end of available observation. All statistical tests were two-tailed, and *p* < 0.05 was considered statistically significant. Analyses were performed using Stata version 16.1 (StataCorp, College Station, TX, USA).

## 3. Results

### 3.1. Baseline Characteristics of Included Participants

**Figure 1:**
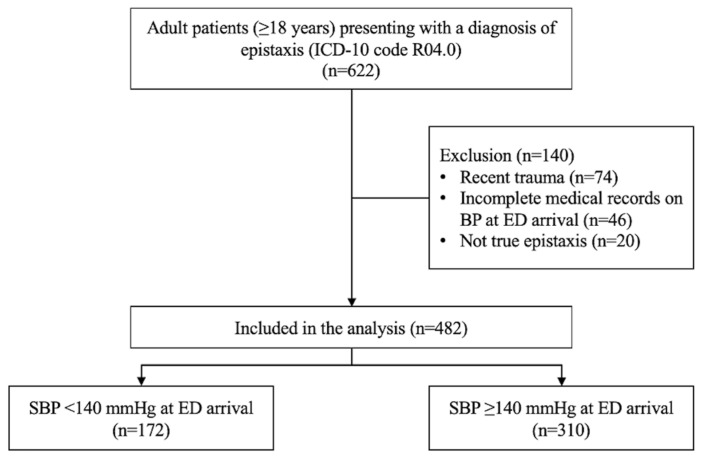
Study Flow Diagram. ED, emergency department; SBP, systolic blood pressure.

During the study period, 622 adult patients (≥18 years) presented to the ED with a diagnosis of epistaxis (ICD-10 code R04.0). A total of 482 patients with non-traumatic epistaxis and recorded systolic blood pressure (SBP) at ED arrival were included in the analysis (Figure 1). Of these, 310 patients (64.3%) presented with SBP ≥ 140 mmHg, and 172 (35.7%) had SBP < 140 mmHg.

Baseline characteristics are summarized in Table 1. Patients with elevated SBP were significantly older (53.0 ± 17.3 vs. 44.4 ± 20.9 years, *p* < 0.001) and more likely to have underlying hypertension (48.7% vs. 19.8%, *p* < 0.001), diabetes (14.2% vs. 5.8%, *p* = 0.008), and dyslipidemia (23.5% vs. 10.5%, *p* = 0.001). DBP and heart rate were also higher among the elevated SBP group (*p* < 0.001 and *p* = 0.009, respectively). No significant differences were observed in sex distribution, cardiovascular disease, heart failure, and chronic kidney disease. The use of anticoagulant and antiplatelet medications was comparable between groups. Bleeding/vascular disorders were more frequent in the lower SBP group (7.6% vs. 1.3%, *p* = 0.001).

Regarding interventions, local hemostatic agents, topical tranexamic acid, mechanical packing, and electrical cauterization were applied with similar frequencies across SBP groups (all *p* > 0.05).

### 3.2. Time to Bleeding Cessation

Overall, the median time to documented bleeding cessation was longer in patients with SBP ≥ 140 mmHg than in those with SBP < 140 mmHg (39 vs. 23 min). Within 30 min, documented bleeding cessation was observed in 30.2% of the SBP < 140 mmHg group compared with 22.9% of the SBP ≥ 140 mmHg group. By 60 min, approximately 40% in each group had documented bleeding cessation, and by ED discharge, hemostasis was documented in 91% and 94%, respectively.

Bleeding cessation was documented in 447 of 482 patients (92.7%). The remaining 35 patients (7.3%) did not have documented hemostasis before ED discharge and were treated as right-censored observations at the time of their last recorded assessment.

### 3.3. Time-to-Event Analysis

This event rate exceeded the 85% event proportion assumed in the original sample size calculation. Cox proportional hazards regression is shown in Table 2. In univariable analysis, SBP ≥ 140 mmHg was associated with a lower, though not statistically significant, hazard of documented bleeding cessation (HR 0.83, 95% CI 0.61–1.13, *p* = 0.233). After multivariable adjustment for demographics, comorbidities, medications, and ED interventions, elevated SBP remained significantly associated with a lower hazard of documented bleeding cessation (HR 0.71, 95% CI 0.52–0.98, *p* = 0.040). Mechanical packing was also independently associated with a lower hazard of documented bleeding cessation (adjusted HR 0.72; 95% CI, 0.54–0.95; *p* = 0.021), reflecting longer times to hemostasis. In contrast, electrical cauterization was associated with a higher hazard of documented bleeding cessation (adjusted HR 2.17; 95% CI, 1.19–3.96; *p* = 0.011). Other covariates, including underlying diseases such as hypertension, use of anticoagulants or antiplatelet agents, and local hemostatic agents, were not significantly associated with time to bleeding control.

An exploratory categorical analysis showed a graded association between higher SBP levels and delayed documentation of bleeding cessation (Supplementary Table S1). Compared with SBP < 140 mmHg, patients with SBP 140–159 mmHg had an adjusted HR of 0.67 (95% CI 0.47–0.97), while those with SBP ≥ 180 mmHg had an adjusted HR of 0.56 (95% CI 0.37–0.85). The association for SBP 160–179 mmHg was directionally similar but did not reach statistical significance.

**Figure 2:**
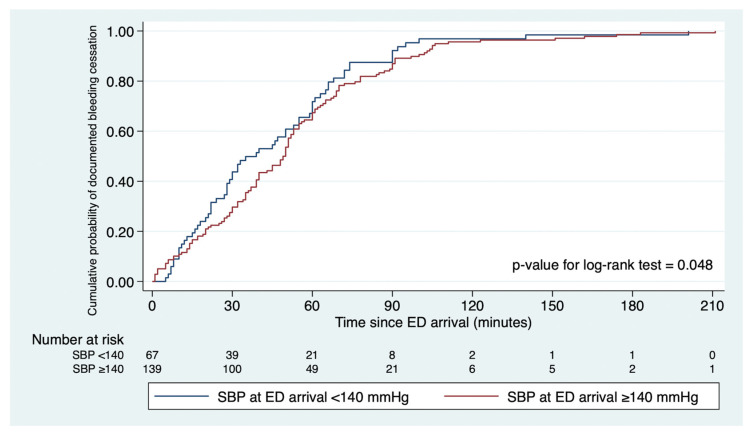
Cumulative probability of documented bleeding cessation stratified by systolic blood pressure (SBP) at emergency department (ED) arrival (<140 vs. ≥140 mmHg).

In the Kaplan–Meier analysis, patients with SBP ≥ 140 mmHg had a longer median time to documented bleeding cessation than those with SBP < 140 mmHg (39 vs. 23 min; log-rank *p* = 0.048; Figure 2). The proportional hazards assumption was tested using Schoenfeld residuals, and no violation was detected (global test, *p* = 0.63).

## 4. Discussion

In this retrospective cohort, elevated systolic BP at ED arrival was associated with a longer time to documented bleeding cessation in adult non-traumatic epistaxis. The association persisted after adjustment for demographic factors, comorbidities, medications, and ED interventions. These findings suggest that arrival SBP may reflect underlying physiological or bleeding-related factors associated with a more prolonged clinical course.

Interpreting triage BP in acute care settings is inherently challenging. A single systolic BP measurement at ED arrival often reflects acute sympathetic activation from anxiety, pain, or active bleeding rather than chronic hypertension [18]. Prior studies have demonstrated wide variability in the correlation between ED BP values and underlying hypertensive status, and misclassification is well recognized [19,20]. Nevertheless, triage systolic BP is consistently and rapidly obtained in routine care and may capture important physiological responses that correspond to bleeding severity or patient distress, thereby offering pragmatic value in early risk stratification [21]. Accordingly, elevated triage SBP should be interpreted primarily as a marker of physiological stress or illness severity rather than definitive evidence of chronic hypertension. Whether elevated SBP itself contributes to delayed hemostasis or merely reflects underlying bleeding severity, patient distress, or sympathetic activation cannot be determined from the present study.

Our findings align with previous literature reporting that higher BP or hypertension history is associated with more persistent bleeding or greater need for intervention [3,8,14]. However, other studies have found no independent association after adjustment for confounders [7,14]. By using time-to-event analysis rather than binary endpoints, our study demonstrates that elevated systolic BP may not determine whether bleeding can ultimately be controlled but may be associated with how long bleeding persists. In an exploratory supplementary analysis using four clinically relevant SBP categories, patients with SBP 140–159 mmHg and SBP ≥ 180 mmHg demonstrated significantly lower hazards of documented bleeding cessation compared with those with SBP < 140 mmHg. Although the study was not originally designed to evaluate dose–response relationships, these findings suggest that progressively elevated SBP may be associated with a more prolonged bleeding course and provide additional support for the robustness of the primary findings. This distinction is clinically relevant because prolonged bleeding contributes to increased discomfort, use of ED resources, monitoring demands, and extended length of stay [22,23,24]. This interpretation should be considered in the context of outcome measurement. Because our outcome was based on documented rather than continuously observed hemostasis, the reported median times should be interpreted as estimates of clinically recognized bleeding cessation rather than exact physiological bleeding duration.

Several mechanisms may explain the observed association. Higher systolic BP may increase local hydrostatic pressure [25] and contribute to clot disruption or impaired stability [26], although available data on this mechanism remain limited and inconsistent. Alternatively, elevated systolic BP may reflect more severe bleeding, greater patient distress, or other unmeasured factors such as posterior bleeding location [4,27]. Patients with higher systolic BP in our cohort were also older and more comorbid, which may have influenced mucosal fragility or healing [28], although these factors were adjusted for in the models. Importantly, this study was not designed to determine whether acute BP lowering improves bleeding control, and no causal inference should be drawn regarding therapeutic BP management [7,8]. Elevated triage SBP should therefore be interpreted as a prognostic marker rather than a therapeutic target. Consistent with existing guidelines and expert reviews, our findings should not be interpreted as supporting routine acute antihypertensive therapy solely to accelerate hemostasis. In fact, unnecessary blood pressure reduction during active bleeding may expose patients to potential harm without established benefit. Whether adjunctive short-term BP optimization improves hemostasis warrants prospective evaluation. An alternative explanation is that elevated SBP does not directly influence bleeding duration but instead serves as a readily measurable indicator of physiological stress, anxiety, pain, or bleeding severity.

Associations with treatment modalities should be considered exploratory and interpreted with substantial caution because treatment selection was not randomized and was strongly influenced by clinician judgment and bleeding characteristics. The association between mechanical packing and longer time to documented bleeding cessation likely reflects confounding by indication rather than a detrimental effect of packing itself. In routine emergency care, packing is generally reserved for patients with more persistent, diffuse, or clinically significant bleeding after initial conservative measures have failed. Consequently, patients receiving packing likely represent a subgroup with more severe epistaxis and a lower baseline probability of rapid hemostasis. Furthermore, our dataset captured whether packing was performed but did not reliably record the precise timing of placement, whether packing was preceded by manual compression or topical therapy, the duration of packing retention, or whether hemostasis was assumed immediately after placement [29]. Conversely, electrical cauterization was associated with faster control of bleeding, which may reflect its use in selected patients with visible, localized bleeding sources that are inherently more amenable to definitive treatment [7]. Therefore, these findings should be interpreted as associations with clinical management patterns rather than estimates of treatment effectiveness. Indeed, the observed associations may primarily reflect underlying differences in bleeding severity, bleeding localization, and visibility of the bleeding source rather than causal effects of the interventions themselves. Consequently, no conclusions regarding the comparative efficacy of these treatments should be drawn from these findings. Future prospective studies incorporating detailed treatment sequencing and standardized assessment intervals are needed to better characterize the relationship between specific interventions and time to hemostasis.

Several limitations should be acknowledged. First, the retrospective design relies on clinician documentation, and the time to document bleeding cessation reflects the timing of the recorded assessment rather than continuous physiological monitoring; documentation delays or workflow constraints may introduce measurement error.

Importantly, the outcome represents the time to documented confirmation of bleeding cessation rather than the exact physiological time at which bleeding stopped. Because bleeding status was assessed intermittently during routine clinical care, the observed event time may have been influenced by the frequency of reassessment, staffing patterns, workflow delays, and documentation practices. In addition, clinicians may have varied in how frequently they reassessed patients and when they documented hemostasis. Although we suspect that any resulting measurement error was largely non-differential with respect to SBP status, we could not directly evaluate this assumption because reassessment intervals and documentation behaviors were not systematically recorded. Consequently, the direction and magnitude of any resulting bias remain uncertain.

Second, the exposure (triage systolic BP) was based on a single, non-standardized measurement with device type, patient position, and measurement timing unavailable; this could lead to misclassification. Third, key clinical variables, including bleeding site (anterior vs. posterior), laterality, estimated blood loss, rebleeding before disposition, provider expertise, and use or timing of analgesics, anxiolytics, intravenous fluids, or antihypertensives, were not consistently available and may confound associations. In addition, the study period spanned multiple years and involved numerous clinicians and care teams. Variability in provider experience, procedural technique, and clinical decision-making may have influenced both management strategies and documentation practices. In particular, posterior epistaxis is often associated with more difficult bleeding control and may occur more frequently among patients with elevated blood pressure. Therefore, part of the observed association between SBP and delayed bleeding cessation may reflect unmeasured differences in bleeding severity rather than a direct effect of blood pressure itself.

In addition, the timing and sequence of epistaxis interventions (e.g., manual compression, topical therapy, packing, and cauterization) were not consistently documented, limiting interpretation of treatment-related associations. Fourth, dichotomizing systolic BP at 140 mmHg simplifies interpretation but results in information loss and limits the assessment of dose–response or non-linear relationships. Fifth, mechanical packing and cautery decisions were clinician-driven and highly susceptible to confounding by indication. Consequently, the observed associations may reflect differences in bleeding severity and treatment selection rather than true treatment effects. Finally, as a single-center study in a tertiary ED in Northern Thailand, generalizability to other practice environments may be limited.

Future research should evaluate BP as a continuous predictor using flexible modeling, incorporate granular bleeding characteristics and treatment timing, validate hemostasis measurement with more objective or standardized methods, and examine whether specific care pathways or BP-informed triage strategies can improve patient flow and resource allocation. Prospective or multicenter studies would help clarify the clinical utility of triage BP in the management of ED epistaxis.

## 5. Conclusions

In this large, 11-year ED cohort, elevated systolic blood pressure at arrival was independently associated with a longer time to documented bleeding cessation in adults with non-traumatic epistaxis. While triage systolic BP should not be interpreted as a causal or directly modifiable factor, important measures of epistaxis severity, including bleeding location and blood loss, were unavailable, and residual confounding remains possible. Therefore, the observed association should be interpreted as prognostic rather than causal. These findings should not be interpreted as evidence supporting routine acute antihypertensive therapy for epistaxis. Future prospective and multicenter studies incorporating detailed bleeding characteristics, standardized BP measurements, and continuous modeling approaches are needed to further clarify the clinical utility of triage systolic blood pressure in patients presenting with epistaxis.

## Figures and Tables

**Table 1:** Baseline characteristics of patients with non-traumatic epistaxis stratified by systolic blood pressure on ED arrival.

Variable	Total (*n* = 482)	SBP ≥ 140 mmHg (*n* = 310)	SBP < 140 mmHg (*n* = 172)	STD
**Demographics**				
Age, years, mean ± SD	49.9 ± 19.1	53.0 ± 17.3	44.4 ± 20.9	0.449
Age ≥ 65 years, *n* (%)	130 (27.0)	89 (28.7)	41 (23.8)	0.111
Male sex, *n* (%)	318 (66.0)	211 (68.1)	107 (62.2)	0.123
**Vital signs at triage**				
SBP, mmHg, mean ± SD	149.5 ± 27.3	165.0 ± 20.1	121.5 ± 11.9	2.629
DBP, mmHg, mean ± SD	90.5 ± 19.9	98.0 ± 19.0	77.1 ± 13.2	1.276
Heart rate, bpm, mean ± SD	92.5 ± 18.9	94.0 ± 18.6	90.0 ± 19.1	0.211
Temperature, °C, mean ± SD	36.7 ± 0.6	36.7 ± 0.6	36.7 ± 0.6	−0.005
Oxygen saturation, %, mean ± SD	97.8 ± 2.3	97.6 ± 2.2	98.1 ± 2.5	−0.214
**Comorbidities**				
Hypertension, *n* (%)	185 (38.4)	151 (48.7)	34 (19.8)	0.639
Diabetes mellitus, *n* (%)	54 (11.2)	44 (14.2)	10 (5.8)	0.281
Dyslipidemia, *n* (%)	91 (18.9)	73 (23.5)	18 (10.5)	0.353
Cerebrovascular disease, *n* (%)	25 (5.2)	15 (4.8)	10 (5.8)	−0.043
Cardiovascular disease, *n* (%)	23 (4.8)	14 (4.5)	9 (5.2)	−0.033
Heart failure, *n* (%)	35 (7.3)	20 (6.5)	15 (8.7)	−0.086
Chronic kidney disease, *n* (%)	26 (5.4)	20 (6.5)	6 (3.5)	0.136
COPD, *n* (%)	4 (0.8)	3 (1.0)	1 (0.6)	0.044
Malignancy, *n* (%)	28 (5.8)	13 (4.2)	15 (8.7)	−0.185
Bleeding/vascular disorders, *n* (%)	17 (3.5)	4 (1.3)	13 (7.6)	−0.308
**Medications**				
Anticoagulant use, *n* (%)	37 (7.7)	22 (7.1)	15 (8.7)	−0.060
Antiplatelet use, *n* (%)	69 (14.3)	47 (15.2)	22 (12.8)	0.068
Inhaled corticosteroid use, *n* (%)	8 (1.7)	5 (1.6)	3 (1.7)	−0.010
History of epistaxis within 72 h, *n* (%)	182 (37.8)	114 (36.8)	68 (39.5)	−0.057
**Interventions in ED**				
Local hemostatic agents, *n* (%)	101 (21.0)	64 (20.6)	37 (21.5)	−0.021
Topical tranexamic acid, *n* (%)	41 (8.5)	21 (6.8)	20 (11.6)	−0.168
Mechanical packing, *n* (%)	172 (35.7)	113 (36.5)	59 (34.3)	0.045
Electrical cauterization, *n* (%)	6 (1.2)	4 (1.3)	2 (1.2)	0.012

Abbreviations: COPD, chronic obstructive pulmonary disease; DBP, diastolic blood pressure; ED, emergency department; SBP, systolic blood pressure; SD, standard deviation; STD, standardized difference. Absolute STD values < 0.10 were considered indicative of negligible between-group imbalance.

**Table 2:** Cox proportional hazards regression for predictors of documented bleeding cessation (*n* = 482; events = 447).

Variable	Univariable HR (95% CI)	*p*-Value	Multivariable HR (95% CI)	*p*-Value
SBP ≥ 140 mmHg	0.83 (0.61–1.13)	0.233	0.71 (0.52–0.98)	0.040
Age (per year)	1.00 (0.99–1.01)	0.700	1.00 (0.99–1.01)	0.859
Male sex	1.21 (0.91–1.61)	0.192	1.29 (0.95–1.75)	0.103
Hypertension	0.95 (0.72–1.27)	0.745	1.15 (0.85–1.57)	0.369
Anticoagulant use	0.95 (0.63–1.44)	0.810	1.04 (0.61–1.77)	0.878
Antiplatelet use	0.97 (0.64–1.47)	0.892	0.83 (0.51–1.38)	0.478
Bleeding disorders	0.74 (0.51–1.07)	0.112	0.52 (0.27–0.98)	0.043
Vascular disorders	0.58 (0.33–1.00)	0.050	0.59 (0.29–1.20)	0.147
Recent epistaxis within 72 h	1.04 (0.78–1.38)	0.815	0.91 (0.67–1.23)	0.531
Inhaled corticosteroid use	1.22 (0.63–2.37)	0.555	1.43 (0.70–2.90)	0.324
Local hemostatic agents	0.79 (0.60–1.04)	0.091	0.83 (0.63–1.09)	0.187
Mechanical packing	0.71 (0.54–0.92)	0.011	0.72 (0.54–0.95)	0.021
Electrical cauterization	1.98 (1.59–2.47)	<0.001	2.17 (1.19–3.96)	0.011

Abbreviations: HR, hazard ratio; SBP, systolic blood pressure. A total of 447 documented bleeding cessation events occurred among 482 included patients. Hazard ratios were estimated using Cox proportional hazards regression.

## Data Availability

The raw data supporting the conclusions of this article will be made available by the authors on request.

## Supplementary Materials

The following supporting information can be downloaded at: https://www.mdpi.com/article/10.3390/jcm15145535/s1, Table S1: Cox proportional hazards regression for predictors of documented bleeding cessation.

## Abbreviations

The following abbreviations are used in this manuscript:

ED	Emergency department
SBP	Systolic blood pressure
HR	Hazard ratio
BP	Blood pressure
CI	Confidence interval
