# Supplementary material for: Association Between Systolic Blood Pressure at Emergency Department Arrival and Time to Bleeding Cessation in Adult Non-Traumatic Epistaxis: A Retrospective Time-to-Event Analysis
Source: J Clin Med. 2026 Jul 15;15(14):5535. doi: 10.3390/jcm15145535 (PMC13412800; doi:10.3390/jcm15145535)
Supplement: Supplementary file 1 [file jcm-15-05535-s001.zip › jcm-4393818-supplementary.pdf]

Supplementary Materials

Table S1. Cox proportional hazards regression for predictors of documented bleeding cessation.

Variable	Univariable HR (95% CI)	*p*-value	Multivariable HR (95% CI)	*p*-value
SBP on arrival				
SBP <140 mmHg	Ref	-	Ref	-
SBP 140-159 mmHg	0.86 (0.62-1.21)	0.401	**0.67 (0.47-0.97)**	**0.032**
SBP 160-179 mmHg	0.83 (0.52-1.32)	0.437	0.78 (0.48-1.28)	0.327
**SBP >180 mmHg**	**0.64 (0.43-0.94)**	**0.022**	**0.56 (0.37-0.85)**	**0.006**
Age (per year)	1.00 (0.99-1.01)	0.700	1.00 (0.99-1.01)	0.949
Male sex	1.21 (0.91-1.61)	0.192	1.24 (0.89-1.73)	0.197
Hypertension	0.95 (0.72-1.27)	0.745	1.21 (0.89-1.65)	0.218
Anticoagulant use	0.95 (0.63-1.44)	0.810	1.03 (0.61-1.75)	0.911
Antiplatelet use	0.97 (0.64-1.47)	0.892	0.81 (0.48-1.36)	0.425
Bleeding disorders	0.74 (0.51-1.07)	0.112	0.50 (0.26-0.95)	0.035
Vascular disorders	0.58 (0.33-1.00)	0.050	0.54 (0.25-1.16)	0.114
Recent epistaxis within 72h	1.04 (0.78-1.38)	0.815	0.90 (0.67-1.22)	0.500
Inhaled corticosteroid use	1.22 (0.63-2.37)	0.555	1.43 (0.69-2.98)	0.335
Local hemostatic agents	0.79 (0.60-1.04)	0.091	0.84 (0.64-1.09)	0.191
**Mechanical packing**	**0.71 (0.54-0.92)**	**0.011**	**0.70 (0.52-0.95)**	**0.023**
Electrical cauterization	**1.98 (1.59-2.47)**	**<0.001**	1.88 (0.90-3.93)	0.092

Abbreviations: HR, hazard ratio; SBP, systolic blood pressure.
